# Effectiveness of a novel experimental herbal toothpaste against bacterial consortium associated with dental caries

**DOI:** 10.4317/jced.61356

**Published:** 2024-06-01

**Authors:** Marco Sánchez-Tito, Lidia-Yileng Tay, Francisco Zea-Gamboa, Raúl Cartagena-Cutipa, Alysson Flores-Gómez, Bruno Spigno-Paco, Tania-Coral-Hernandez Cadenas, Ingrit-Elida-Collantes Díaz

**Affiliations:** 1 Faculty of Health Sciences, Universidad Privada de Tacna, Tacna, Peru; 2 Faculty of Stomatology, Universidad Peruana Cayetano Heredia, Lima, Peru; 3 Faculty of Chemistry and Textile Engineering, Universidad Nacional de Ingeniería, Lima, Peru

## Abstract

**Background:**

To evaluate the effectiveness of an experimental toothpaste based on *Hypericum laricifolium* Juss. essential oil against a bacterial consortium associated with dental caries.

**Material and Methods:**

The antibacterial activity of the essential oil was evaluated by the disk diffusion and microdilution tests against *Streptococcus mutans*, *Streptococcus sanguinis* and *Streptococcus salivarius*. Petri dishes were incubated at 37°C for 24 h. An experimental toothpaste was prepared at a concentration of 201.76 mg/mL. The antibacterial activity of the experimental and six commercial toothpastes were evaluated by agar well-diffusion method. Petri dishes were inoculated with a bacterial consortium prepared with the three strains. 80 mg of the toothpastes were placed in the wells and the Petri dishes were incubated at 37°C for 24 h. The inhibition zones were measured with a digital compass. The differences between the pastas were evaluated with the one-way ANOVA test, with a 5% level of significance.

**Results:**

The essential oil was more effective than 0.12% chlorhexidine in inhibiting the growth of *S. mutans* (29.02±1.74 mm) and *S. sanguinis* (21.92±3.43 mm), being more moderate for *S. salivarius* (17.66±1.11 mm) . In MBC tests, the EO showed complete inhibition of the growth of *S. mutans* at a concentration of 5% (50.44 mg/mL), 10% (100.88 mg/mL) for *S. sanguinis* and 2.5% (25.22 mg/mL) for *S. salivarius*. The experimental toothpaste was effective in inhibiting the growth of the bacterial consortium (31.88±66 mm), having a similar performance to Total Dent, Colgate Total 12, Kolynos® Herbal and Colgate® Herbal (*p*> 0.05).

**Conclusions:**

The development of an experimental paste based on H. laricifolium Juss. essential oil (0.28% v/v) showed an important antibacterial activity similar to commercial toothpastes against a bacterial consortium of *S. mutans*, *S. sanguinis* and *S. salivarius*.

** Key words:**Antibacterial agents, toothpastes, medicinal plants, Streptococcus mutans, Streptococcus sanguinis, Streptococcus salivarius.

## Introduction

Dental caries continues to be the main disease of the oral cavity worldwide, it has been estimated that untreated dental caries affects 2.5 billion adults and 573 million children around the world (1).

Dental caries is a dynamic, complex disease, and is the result of dysbiotic changes in the oral biofilm mediated by the frequency of consumption of fermenTable sugars and carbohydrates (2). Furthermore, its progression is controlled by an intricate relationship of factors between the host and the oral microbial flora (3).

*Streptococcus mutans* is recognized as the most important pathogen in the generation of dental caries (4). *S. mutans* colonizes the hard tissues of the tooth, producing an alteration in the homeostatic balance of the dental biofilm, inducing demineralization of the tooth surface with loss of calcium and phosphate ions, and consequently forming carious lesions in pits, fissures and on flat surfaces of the tooth (5).

Lamont *et al*., point out that there is interspecies competition that is capable of altering the oral microbiota before carious lesions appear (6). Thus, Streptococcus salivarius has been identified as a potential agent in the formation of dental caries (7). S. salivarius is part of the commensal flora, however it has been isolated in areas close to deep carious lesions, so it can be considered an indicator of cariogenic activity, especially in patients with low levels of *S. mutans* (8). *Streptococcus sanguinis* is a primary colonizer and collaborates in the attachment of successor organisms, playing an important role in the development of the biofilm (9).

Various strategies to prevent the formation and progression of dental caries have been proposed, which include education for correct oral hygiene, control of eating habits, use of fluorides and antimicrobial agents (10). In recent years, there is an increased interest in the study and use of natural antibacterial agents that reduce the possibility of generating antibiotic resistance.

The use of plants for the treatment of various diseases has been recorded since ancient times, and is part of the folklore and traditional knowledge of the people (11). *H. laricifolium* Juss. It is a species that is distributed in the Andean region of South America and has been traditionally used as an anti-inflammatory, antidepressant and antibiotic (12). Phytochemical studies of the aerial parts of this plant have revealed the presence of phenolic acids, flavonoids, triterpenoids, xanthones and acylphloroglucinols derivatives (12-14).

It is known that the identification of chemical compounds of plant derivatives such as essential oils and extracts varies according to different factors, such as the anatomical and physiological characteristics of the plants, habitat conditions, climatic changes, among others (15).

Essential oils derived from medicinal plants could be used for the formulation of various antibacterial agents such as gels and toothpastes, given the important antibacterial properties of their chemical compounds (16,17).

Therefore, the aim of this study was to the evaluate the effectiveness of the essential oil obtained from *H. laricifolium* Juss. as an active product in the formulation of an experimental toothpaste against a bacterial consortium associated with dental caries.

## Material and Methods

-Study design and sample size calculation

This *in vitro* experimental study was approved by the Institutional Ethics Committee of the Faculty of Health Sciences of the Private University of Tacna with registration FACSA-CEI/020-05-2023.

The calculation of the sample for the antibacterial sensitivity test of the toothpastes was carried out with the G*Power 3.1.3 program (Heinrich Heine Universität, Düsseldorf, Germany), considering the multiple comparison between groups (one-way ANOVA with fixed effects). An effect size of 0.912 was considered, calculated from the standard deviation within the groups of a previous study (16), an α error probability of 0.05 and a power of 0.8. A total of 28 repetitions were calculated (with a power of 0.91). For this study, the number of repetitions was increased to 5 per group (n=35), increasing the power to 0.97.

-Extraction and preparation of the essential oil 

Aerial parts of *Hypericum Laricifolium* Juss. were obtained from the community of Llacubamba, Parcoy District, Pataz Province, La Libertad Department, Peru. The essential oil (EO) was obtained by the hydrodistillation method for 4 hours using a Clevenger apparatus. The EO obtained was dried with sodium sulfate and stored at -20°C until use (18). EO was weighed to calculate its yield in relation to biomass. Dissolutions of the EO were prepared at concentration of 80%, 60%, 40%, 20%, 10%, 5% and 2.5%. Dimethyl sulfoxide - DMSO (Loba Chemie Pvt. Ltd. Mumbai, India) was used to prepare the dissolutions (19). 0.5 mL of each dissolution was prepared and stored in correctly labeled cryovials until use.

-Activation of bacterial strains

Strains of *Streptococcus mutans* ATCC® 25175TM, *Streptococcus sanguinis* ATCC® 10556TM and *Streptococcus salivarius* ATCC® 13419TM were used. The strains were sown in Petri dishes (90 x 15 mm) containing Brain-Heart Agar (Liofilchem®, Abruzos, TE, Italy) and incubated at 37°C for 48 h. Using a sterile loop, some colonies were taken to a tube containing 5 mL of Brain-Heart Infusion Broth (Liofilchem®, Abruzos, TE, Italy) and incubated at 37°C for 7 h, until the exponential growth phase. Bacterial suspensions were adjusted at a concentration of 1.5x108 corresponding to the McFarland Standard 0.5.

-Disk diffusion assay

Sterile filter paper discs of 6 mm diameter impregnated with 10 µL of the different dissolutions of the EO were used. The discs were placed in Petri dishes containing Brain-Heart Agar inoculated with strains of *S. mutans* ATCC® 25175TM, *S. sanguinis* ATCC® 10556TM and *S. salivarius* ATCC® 13419TM individually. The dishes were incubated anaerobically using a GasPack CO2 system at 37 °C for 24 h. Bacterial sensitivity was evaluated by measuring the diameter of the inhibition zones with a digital compass . The assay was performed in triplicate.

-Microdilution assay

To determine the minimal bactericidal concentration (MBC), the microdilution assay was performed following the recommendations of the Clinical and Laboratory Standards Institute (20). For the assay, 96-well microplates with round bottoms were used. Columns A to H and rows 1, 3 and 5 were used for the EO dissolutions and the wells in columns I and J were used for the positive and negative controls. 190 µL of the bacterial suspension was added to each well, then 10 µL of each dissolution of the EO was added to the corresponding well (18,19). Chlorhexidine at 0.12% (Perio.Aid®, Dentaid, Barcelona, Spain) and DMSO were used as positive and negative control, respectively. The microplates were incubated at 37°C for 24 h under anaerobic conditions. Then, 2 µL from each well were transferred to a Petri dish containing Brain-Heart Agar and incubated at the same conditions. Subsequently, bacterial growth was evaluated through colony-forming unit (CFU) counting, considering a seeding dilution of 10-20. The assay was carried out for each strain.

-Experimental toothpaste formulation

The ingredients and quantities used for the formulation of the experimental toothpaste are presented in  Table 1. The ingredients were weighed on a 4-digit digital scale. 40 mL of distilled water was poured in a beaker. Calcium carbonate, carboxymethyl cellulose, methylparaben and propylparaben were mixed in a sterile beaker, this mixture was slowly incorporated into the initial beaker maintaining a constant stirring for 15 min at 40 °C until its homogeneous mixture. In another beaker with 30 mL of distilled water, sodium lauryl sulfate, sorbitol, sodium saccharine, menthol and titanium dioxide were incorporated. This second mixture was maintained at a temperature of 40 °C before being poured into the first beaker. Finally, the essential oil was added and stirring was maintained for another 10 min to ensure homogenization of the toothpaste (21). The concentration of the EO was determined based on the previous results of the MBC, using a concentration of 201.76 mg/mL, corresponding to the 20% EO dissolution. All reagents were purchased from IDSA EIRL (Lima, Peru). For this study, six commercial toothpastes were used to compare the antibacterial activity of the experimental toothpaste. The composition of the commercial toothpastes is listed in  Table 2.

-Agar-well diffusion assay

A bacterial consortium was prepared from the initial suspensions of *S. mutans* ATCC® 25175TM, *S. sanguinis* ATCC® 10556TM and *S. salivarius* ATCC® 13419TM. The mixture was homogenized in a vortex mixer for 1 min. Petri dishes were prepared with Brain-Heart Agar that were inoculated with a suspension adjusted to the 0.5 McFarland Standard of the bacteria consortium. Wells were created on the agar of each Petri dish using a 6 mm diameter steel biopsy punch. 80 mg of each toothpaste was added to each well (16). The Petri dishes were incubated anaerobically at 37 °C for 24 h. The bacterial growth inhibition zones were measured with a digital compass. A total of 5 repetitions were performed for each toothpaste.

- Statistical analysis

The data were analyzed with the Stata® software version 17 (StataCorp LP, College Station, TX, USA). The statistical analysis included descriptive statistics such as measures of central tendency and dispersion. The assumptions of normality and homogeneity of variances for the bacterial growth inhibition values were evaluated with the Shapiro-Wilk and Bartlett tests, respectively. One-way ANOVA test was performed to verify the existence of differences between the toothpastes. Subsequently, the Scheffé test was used for multiple comparisons. The effect size was also calculated through the eta squared estimator. The significance level was adjusted to 5% for all tests.

## Results

**Figure 1:**
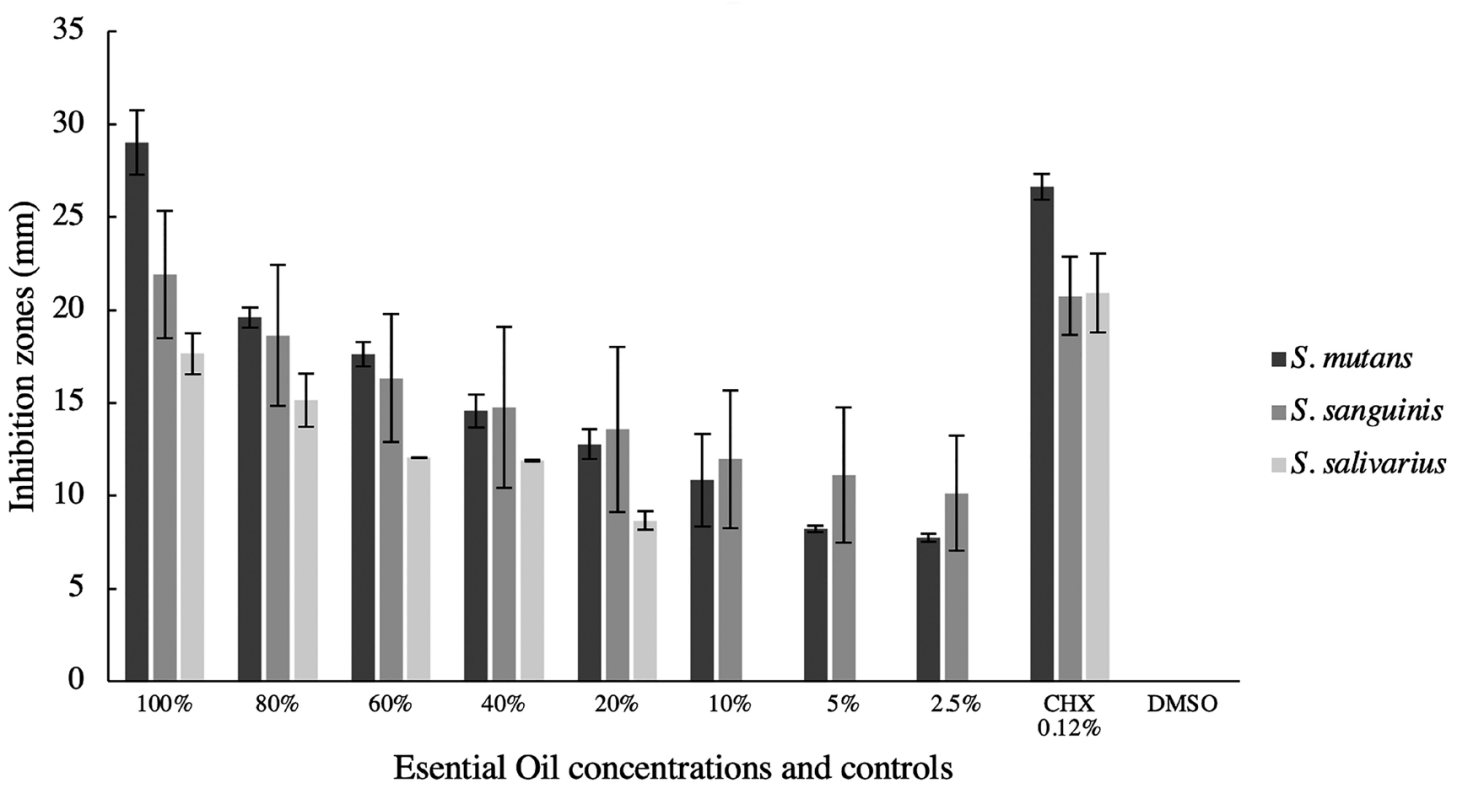
Inhibition zones in mm of the essential oil dissolution for *S. mutans*, *S. sanguinis* and *S. salivarius*.

An EO yield of 0.25% was observed, with a density of 1008.8 mg/mL. The results of the disk diffusion assay showed that 100% EO was more effective than 0.12% chlorhexidine in inhibiting the growth of *S. mutans* (29.02±1.74 mm) and *S. sanguinis* (21.92±3.43 mm), being more moderate for *S. salivarius* (17.66±1.11 mm). Concentrations greater than 20% showed inhibition zones above 12 mm for the three bacterial strains (Fig. 1).

In MBC tests, *H. Laricifolium* Juss. EO showed complete inhibition of the growth of *S. mutans* at a concentration of 5% (50.44 mg/mL), 10% (100.88 mg/mL) for *S. sanguinis* and 2.5% (25.22 mg/mL) for *S. salivarius*, ( Table 3).

**Figure 2:**
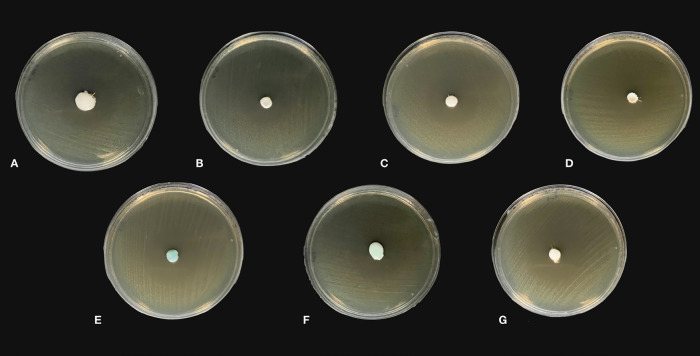
Antibacterial activity of toothpastes against bacterial consortium. A: Experimental toothpaste, B: Vitis Orthodontic, C: Total Dent, D: Colgate® Total 12, E: Kolynos® Herbal, F: Colgate Herbal®, G: Oral B®.

The experimental toothpaste formulation had a final concentration of 0.28% of active agent.  Table 4 shows the measurements of the inhibition zones generated by the different toothpastes against the bacterial consortium. The results showed significant differences between the toothpastes (*p* < 0.05). Furthermore, it was observed that the highest antibacterial activity was for Oral B® (34.81±0.45 mm), although no significant differences were reported with the rest of the toothpastes (*p* > 0.05), except for Vitis Orthodontic and Colgate® Total 12 (*p* < 0.05). Multiple comparisons showed that the experimental paste based on EO of *H. Laricifolium* Juss. EO was effective in inhibiting the growth of the bacterial consortium (31.88±66 mm), having a similar performance to Total Dent, Colgate Total 12, Kolynos® Herbal and Colgate® Herbal (*p* > 0.05). The estimated effect size (ŋ2 = 0.79) indicates that 79% of the variability observed in the bacterial growth inhibition zones can be attributed to differences in the constituents of the toothpastes. The details of the inhibition zones generated by the toothpastes are shown in Figure 2.

## Discussion

Various oral hygiene methods have been introduced to reduce the accumulation of biofilm on the surface of the teeth and consequently reduce the risk of formation of caries lesions. Tooth brushing is the main mechanical procedure to eliminate biofilm formation (10); However, it is known that it is not enough to reduce the formation of dental caries. Consequently, the incorporation of antibacterial agents in toothpastes has proven to be an important chemotherapeutic mechanism that, together with a correct brushing technique, can be effective in reducing the rate of caries formation (22).

Most commercial toothpastes have incorporated fluoride and triclosan as active agents due to their high effectiveness in reducing bacterial viability (23). However, it has been reported that its prolonged use may have adverse effects (24,25). In this sense, the proposal to use organic agents from medicinal plants is an interesting and viable option for bacterial control associated with various oral pathologies.

Although there are no previous studies that have reported the effect of *H. laricifolium* Juss. EO on bacteria associated with the formation of dental caries, other reports have identified that the extracts and EO obtained from the Hypericum genus have important antimicrobial properties (26-28).

In this study, the disk diffusion method was used to determine the antibacterial activity of the essential oil, considering that it is a recommended method to assess the antimicrobial activity of extracts and oils obtained from plants (29). Furthermore, it has been suggested that this method can prevent the volatilization and dispersion of essential oils (16). The results showed that the pure EO was highly effective in inhibiting the growth of *S. mutans* and *S. sanguinis* even more than 0.12% chlorhexidine, presenting somewhat lower activity for *S. salivarius*. When concentrations greater than 40% of the EO were used, inhibition zones greater than 12 mm were obtained for the three bacterial strains, which has been considered an effective measure in other studies carried out with medicinal plant extracts (30).

The high antibacterial activity of *H. laricifolium* juss. EO can be explained due to the great variety of constituents that have been identified in the literature. The presence of limonene, (E)-β-ocimene, (Z)-β-ocimene, α-pinene, verticiol, 3-methyl-nonane, 2-methyl-octane, nonane, among others, has been mainly detected among others (31,32). Regarding the mechanism of antibacterial action, it has been suggested that monoterpenes and alkanes act at the level of the lipid bilayer of the plasma membrane, interfering in the synthesis of some proteins and enzymes on the surface of the membrane, causing structural damage and an increase of permeability, leading to cell death (33,34).

On the other hand, to test the antibacterial activity of toothpastes, the well diffusion method was chosen. This method has been widely recommended in other studies (35), since the inclusion of the well in the agar allows for simple and effective toothpaste volumes (16,17).

In this study, a bacterial consortium was used to test the effectiveness of toothpastes. Monobacterial models have been widely used to recreate enamel demineralization processes, mainly based on *S. mutans*. It is important to recognize that the formation of carious lesions is a highly complex process, where biofilms contain multiple species, so trying to approximate these models is quite relevant (36).

The main results of this study showed that the experimental toothpaste based on *H. laricifolium* Juss. EO had significant antibacterial activity against a bacterial consortium formed by *S. mutans*, *S. sanguinis* and *S. salivarius*. The experimental toothpaste showed similar activity to commercial toothpastes, particularly with Kolynos® Herbal, Colgate® Herbal and Total Dent herbal fresh mint. According to the manufacturers, Kolynos® Herbal contains as active elements commiphora myrrha oil, anthemis nobilis flower oil, salvia officinalis oil, and eucalyptol; while Colgate® Herbal mainly contains limonene; and melissa officinalis flower/leaf/stem oil was the active ingredient for Total Dent herbal fresh mint.

As previously mentioned, the antimicrobial mechanism of the monoterpenes contained in essential oils occurs on the permeability of the membrane, which would explain the similarity in the results obtained against the experimental toothpaste. In general, commercial toothpastes have Sodium fluoride and monofluorophosphate as their main active agent at different concentrations.

In the literature there are no studies that incorporate the essential oil of *H. laricifolium* Juss. in a toothpaste, hiding comparison of results. However, other studies incorporated essential oils from medicinal plants in toothpastes, demonstrating their high effectiveness against *S. mutans* and other species. De Oliveira *et al*. demonstrated that toothpastes containing essential oils of clove, oregano, thyme and cinnamon at low concentrations were able to completely disrupt *S. mutans* biofilms (17). Karadağlıoğlu *et al*. evaluated the combination of toothpastes with essential oils of oregano and cinnamon. Their results showed that the pastes were effective in inhibiting the growth of *S. mutans* (16).

Considering the limitations of this study, it is necessary to develop additional studies to verify the effectiveness of the experimental toothpaste using a multispecies biofilm model including a greater number of bacterial strains. As well as its potential cytotoxicity before implementing *in vivo* studies.

## Conclusions

The development of an experimental paste based on *H. laricifolium* Juss. essential oil (0.28% v/v) showed an important antibacterial activity similar to commercial pastes against a bacterial consortium of *S. mutans*, *S. sanguinis* and *S. salivarius*.

## Figures and Tables

**Table 1:** Experimental toothpaste composition.

Ingredient	Quantity	Property
Calcium carbonate	2.5 g	Abrasive
Sodium lauryl sulphate	1.5 g	Surfactant
Sorbitol	2 mL	Humectant
Sodium Carboxy methyl cellulose	2 g	Binding agent
Sodium saccharine	0.3 g	Sweetener
Methylparaben	01 g	Preservative
Propylparaben	0.02 g	Preservative
Titanium dioxide	0.5 g	Opacifier
Menthol	0.2 g	Flavoring agent
Essential oil	200 ul	Active agent
Distilled water	70 mL	Vehicle

**Table 2:** Composition of the commercial toothpastes.

Toothpaste	Company	Composition
Vitis orthodontic	Dentaid S.L., Cerdanyola, Spain	Aqua, sorbitol, silica, glycerin, xanthan gum, titanium dioxide, xylitol, sodium gluconate, sodium lauryl sulfate, sodium fluoride, sodium saccharin, menthone glycerin acetal, aloe barbadensis leaf juice, allantoin, sodium methylparaben, lactic acid, cetylpyridinium chloride, aroma.
Total Dent herbal fresh mint	Total Dent, China	Calcium carbonate, aqua, sorbitol, glycerin, silica, sodium lauryl sulfate, aroma, cellulose gum, sodium monofluoorophosphate 0.76%, sodium fluoride 0.1%, Xanthan gum, sodium saccharin, tetrasodium pyrophosphate, PEG-32, benzyl alcohol, melissa officinalis flower/leaf/stem oil, sodium benzoate.
Colgate®Total 12	Colgate Palmolive, Guanajuato, Mexico	Glycerin, aqua, hydrated silica, sodium lauryl sulfate, aroma, arginine, zinc oxide, cellulose gum, CI 77891, benzyl slvohol, poloxamer 407, zinc citrate, tetrasodium pyrophosphate, xanthan gum, cocamidopropyl betaine, sodium fluoride, sodium sccharin, phosphoric acid, sucralose.
Kolynos®herbal original	Colgate-Palmolive, Guanzhou, China	Calcium carbonate, aqua, sorbitol, sodium lauryl sulfate, hydrated silica, aroma, commiphora myrrha oil, manzanilla – anthemis nobilis flower oil, salvia officinalis oil, eucalyptol, sodium monofluorophosphate, cellulose gum, magnesium aluminium silicate, sodium saccharin, sodium carbonate, benzyl alcohol, sodium bicarbonate, CI 74260, limonene.
Colgate®Herbal original	Colgate Palmolive, Guanzhou, China	Aqua, calcium carbonate, sorbitol, hydrated silica, sodium lauryl sulfate, aroma, sodium monofluorophosphate, cellulose gum, sodium saccharine, Xanthan gum, sodium carbonate, benzyl alcohol, sodium bicarbonate, CI 74260, limonene.
Oral B®	Procter & Gamble, Naucalpan de Juárez, Mexico	Sodium fluoride, aqua, sorbitol, silica, disodium pyrophosphate, sodium laurylsulfate, cellulose gum, aroma, sodium hidroxide, sodium saccharin, carbomer, Xanthan gum, titanium dioxide, eugenol, sodium benzoate, potassium sorbate

**Table 3:** Minimal bactericidal concentration obtained from H. laricifolium essential oil dissolutions evaluated against S. mutans, S. sanguinis and S. salivarius.

Concentration (mg/mL)	S. mutans (CFU)	S. sanguinis (CFU)	S. salivarius (CFU)
100 % (1008.8)	< 3	< 3	< 3
80 % (807.04)	< 3	< 3	< 3
60 % (605.28)	< 3	< 3	< 3
40 % (403.52)	< 3	< 3	< 3
20 % (201.76)	< 3	< 3	< 3
10 % (100.88)	< 3	< 3	< 3
5 % (50.44)	> 300	205	< 3
2.5 % (25.22)	> 300	> 300	< 3
CHX 0.12%	< 3	< 3	< 3
DMSO	> 300	> 300	> 300

CFU: colony-forming unit.

**Table 4:** Toothpastes inhibition zones (mm) against bacterial consortium.

Toothpaste	Mean±SD	Min	Max	P valor^*^	ŋ^2^
Experimental Toothpaste	31.88±0.66^ac^	31.01	32.48	<0.0001	0.79
Vitis Orthodontic	26.69±1.23^b^	25.66	28.69		
Total Dent	34.14±1.85^ac^	32.15	35.94		
Colgate^®^ Total 12	31.37±1.36^a^	29.81	32.81		
Kolynos^®^ Herbal	33.10±0.63^ac^	32.17	33.77		
Colgate^®^ Herbal	33.97±2.62^ac^	31.27	37.39		
Oral B^®^	34.81±0.45^c^	34.28	35.49		

## Data Availability

The datasets used and/or analyzed during the current study are available from the corresponding author.
